# The Role of Metabolites in Acyclovir-Induced Neurotoxicity and Nephrotoxicity

**DOI:** 10.3390/medicines13010006

**Published:** 2026-02-02

**Authors:** Asma Aboelezz, Sherif Hanafy Mahmoud

**Affiliations:** Faculty of Pharmacy and Pharmaceutical Sciences, University of Alberta, Edmonton, AB T6G 2E1, Canada; aboelezz@ualberta.ca

**Keywords:** acyclovir, 9-carboxymethoxymethylguanine (CMMG), nephrotoxicity, neurotoxicity

## Abstract

Acyclovir is an antiviral drug effective against infections caused by herpes simplex and varicella zoster viruses. It is given intravenously to treat serious infections such as herpes encephalitis. High acyclovir concentrations could cause toxicity, observed mainly as nephrotoxicity and, to a lesser extent, neurotoxicity. Acyclovir nephrotoxicity is primarily attributed to the crystallization of acyclovir within the renal tubules, although additional mechanisms may also contribute. However, the mechanism of acyclovir-induced neurotoxicity is unknown. Acyclovir is mainly eliminated from the body through renal excretion; however, around 15–20% of acyclovir is metabolized subsequently by alcohol and aldehyde dehydrogenase to the main metabolite 9-carboxymethoxymethylguanine (CMMG), and around 2% is metabolized by aldehyde oxidase to the minor metabolite, 8-hydroxyl acyclovir. It has been suggested that CMMG levels above 10 µmol/L in the serum and 1 µmol/L in the cerebrospinal fluid are highly associated with neurotoxicity. Studies have shown that there is a potential contribution of CMMG to acyclovir-induced neurotoxicity and of the acyclovir aldehyde to nephrotoxicity. In this narrative review, we approach the topic of acyclovir metabolites and their association with acyclovir toxicity. Moreover, we identify the research gap of the mechanisms by which these metabolites contribute to toxicity.

## 1. Introduction

Herpes viruses cause a wide variety of infections ranging from mild cold sores and genital herpes to serious life-threatening herpes encephalitis [1,2,3,4]. Herpes infections are spread worldwide, with millions of people infected and carrying a significant disease and economic burden [5]. Ensuring safe and effective antiviral treatment is crucial, as around $35 billion was spent globally in 2016 to manage only genital herpes infections [6].

Acyclovir and valacyclovir are among the primary antiviral medications used to treat herpes infections caused by herpes simplex viruses (HSV) and varicella zoster virus (VZV) [7,8]. Acyclovir (9-[(2-hydroxyethoxy)methyl]-9H-guanine) is a nucleoside analog with antiviral activity [9,10]. Once acyclovir is activated, it acts through competitive inhibition of the viral DNA polymerase and subsequently inhibits the DNA replication [11].

Acyclovir is mainly eliminated through the kidney, with a small portion subjected to metabolism. It is metabolized to 9-carboxymethoxymethylguanine (CMMG) and 8-hydroxyl acyclovir, which are pharmacologically inactive. CMMG is formed after sequential steps of metabolism with the formation of a short-lasting intermediate (acyclovir aldehyde). Acyclovir metabolism occurs mainly in the kidney with the involvement of aldehyde oxidase, alcohol dehydrogenase (ADH), and aldehyde dehydrogenase (ALDH) enzymes [12].

Acyclovir could cause toxicity ranging from mild gastrointestinal irritation to more serious nephrotoxicity and neurotoxicity [13]. Acyclovir-induced nephrotoxicity could be attributed to the crystallization of acyclovir in the renal tubules, as it has low solubility [14,15]. Moreover, it has been suggested that it causes direct cell death in the kidney by itself or through the intermediate metabolite acyclovir aldehyde [16,17]. On the other hand, it was also reported that high CMMG levels are associated with neurotoxicity [18]. A possible risk factor that could be associated with acyclovir-induced toxicity is the use of high doses [19], or even standard doses if the patient is not well hydrated or has reduced kidney function. Acyclovir toxicity could be reversed with dose adjustment, adequate hydration, or dialysis [20].

This review examines the possible contributions of acyclovir aldehyde and CMMG to the development of acyclovir-induced nephrotoxicity and neurotoxicity. We summarize the existing evidence of the contribution of acyclovir metabolites to its overall toxicity. Furthermore, we address the current knowledge gap of the mechanisms by which these metabolites exert their toxicity. To ensure a thorough overview of available literature, we searched the Scopus, PubMed, and Embase databases.

## 2. Acyclovir and Its Use

**Figure 1:**
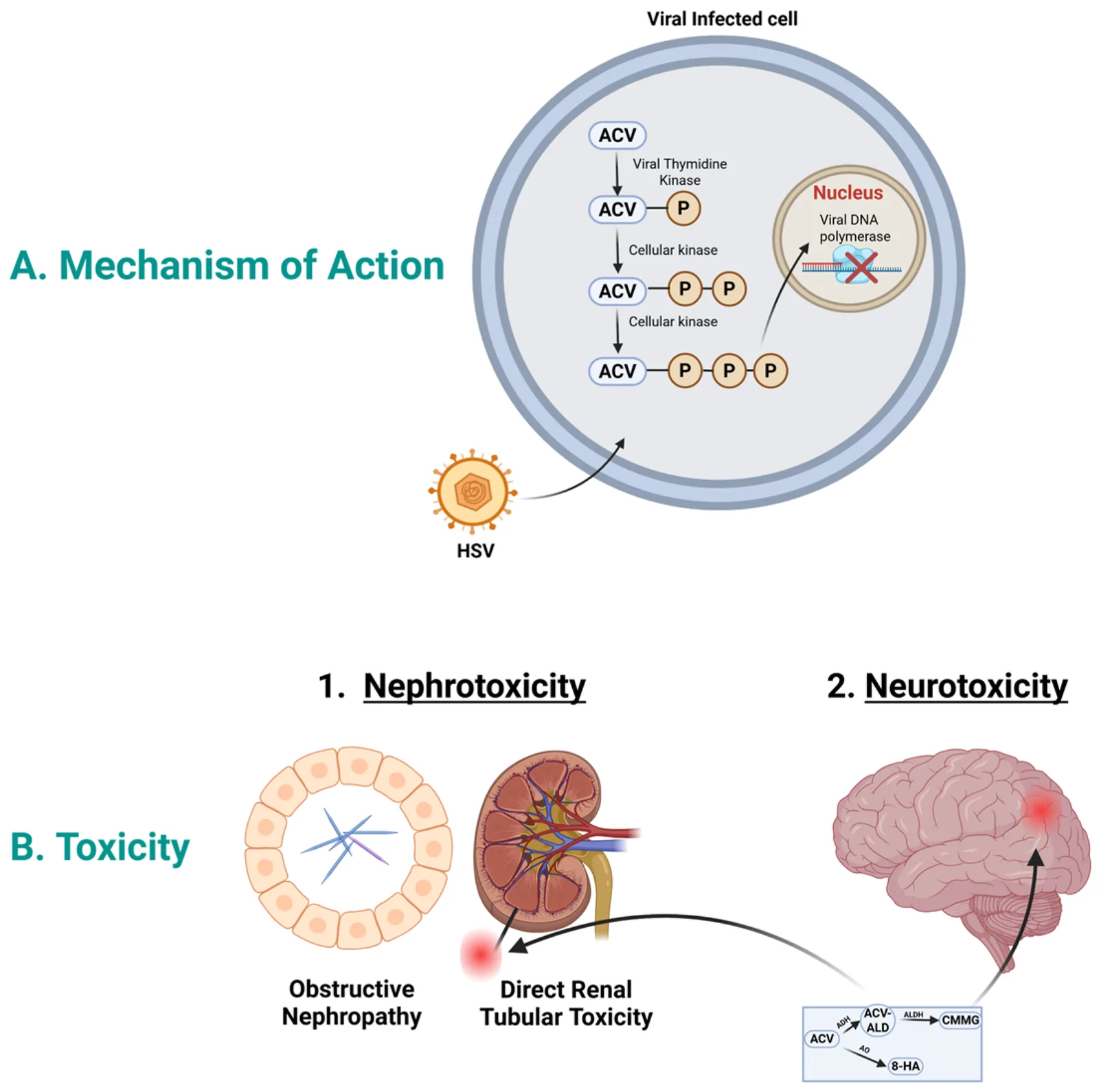
Acyclovir mechanism of action and toxicity. (**A**). Mechanism of action: In HSV infected cells, acyclovir is selectively phosphorylated by viral thymidine kinase into acyclovir monophosphate. Host cell enzymes further convert this into the active acyclovir triphosphate form. This compound moves to the nucleus and acts as a potent inhibitor of viral DNA polymerase, causing DNA chain termination and halting viral replication. (**B**). Acyclovir toxicity: Acyclovir could cause nephrotoxicity and neurotoxicity. Acyclovir aldehyde could play role in nephrotoxicity, while CMMG could play role in neurotoxicity. Abbreviations; ACV, acyclovir; ACV-ALD, acyclovir aldehyde; HSV, herpes simplex virus; CMMG, 9-carboxymethoxymethylguanine; 8-HA, 8-hydroxy acyclovir. Created in BioRender. Aboelezz, A. (2026) https://BioRender.com/xayni5v (accessed on 2 January 2026).

To exert its action, acyclovir initially is activated by the viral thymidine kinase and phosphorylated to acyclovir monophosphate; see Figure 1A. Then, subsequent phosphorylation in the cell by cellular kinase to acyclovir di-phosphate and to acyclovir triphosphate. Acyclovir triphosphate inhibits the viral DNA polymerase, which in turn stops the viral DNA synthesis [21]. Acyclovir triphosphate is structurally similar to the natural building block of DNA, “deoxyguanosine triphosphate”; therefore, it competes for the active site of viral DNA polymerase. This results in the incorporation into the growing viral DNA strand, causing inactivation of the viral DNA polymerase [22].

Various dosage forms are available for acyclovir: oral, intravenous (IV), and topical forms, with each given for specific indications [10]. IV acyclovir is used to treat serious infections such as herpes encephalitis or could be used in immunocompromised patients to treat less serious infections. It is given in a dose of 10 mg/kg intravenously every eight hours to treat herpes encephalitis [23]. Oral acyclovir has poor bioavailability, and its use is limited to less severe infections such as genital herpes or cold sores [24].

The oral bioavailability of acyclovir is very low, ranging from 15 to 30% [25], which renders the oral formulations an unsuitable option in serious infections such as herpes encephalitis or in disseminated herpes infection. Valacyclovir is an oral prodrug that is converted in the body to acyclovir. It is the L-valyl ester of acyclovir, which enhances the absorption of acyclovir in the gastrointestinal tract. Valacyclovir has an enhanced oral bioavailability of 50–70%, which renders the oral availability a preferred choice in many cases [26].

## 3. Acyclovir Pharmacokinetics and Metabolism

### 3.1. Acyclovir Pharmacokinetics

Acyclovir has poor passive absorption in the gastrointestinal tract due to its hydrophilicity. On the other hand, valacyclovir (L-valyl ester of acyclovir) is rapidly absorbed through the gastrointestinal tract via peptide transporters, specifically human peptide transporters 1 in the intestine [27,28]. It undergoes rapid and extensive first-pass metabolism, and it is converted by esterase enzymes to acyclovir [29,30,31]. Valacyclovir is rapidly de-esterified in the intestinal lumen, intestinal wall, and liver. The portion that is de-esterified in the intestinal lumen is excreted in the feces, and the portion that is de-esterified in the intestinal wall and liver reaches the systemic circulation and exerts its action. The enhanced bioavailability of valacyclovir renders it a better option in the outpatient care setting and as an alternative to intravenous acyclovir in some conditions [30].

Acyclovir is mainly distributed in the total body water, as it has a volume of distribution of around 42 L. It has low plasma protein binding of 15% and a half-life of around 2–3 h [32,33]. Around 62 to 91% of acyclovir is eliminated unchanged in the urine [32], and the remaining is metabolized to CMMG and 8-hydroxy acyclovir. Acyclovir has a renal clearance of 248 ± 46 mL/min/1.73 m^2^ [34], which is around three times that of creatinine clearance; therefore, active tubular secretion plays a role in acyclovir clearance with glomerular filtration [35]. Organic anionic transporters (OATs) are involved in the influx of acyclovir to the tubular cells, mainly OAT1 and OAT3 [36]. On the other hand, human breast cancer resistance protein (BCRP) is involved in the efflux of acyclovir to the tubular lumen [37]. Because acyclovir is not transported by the same transporters as creatinine, elevated creatinine levels during acyclovir therapy are a real indication of nephrotoxicity, as they are not competing for the same transporters [38]. Moreover, genetic variations in OAT1, OAT3, and BCRP are known to alter the pharmacokinetics of drugs transported by them [39,40]. To our knowledge, there are no studies evaluating if any genetic variation in these transporters has an impact on the concentration of acyclovir in the tubular cells and, subsequently, if this could have an impact on the metabolism of acyclovir in the tubular cells.

### 3.2. Major Metabolites and Their Formation

**Figure 2:**
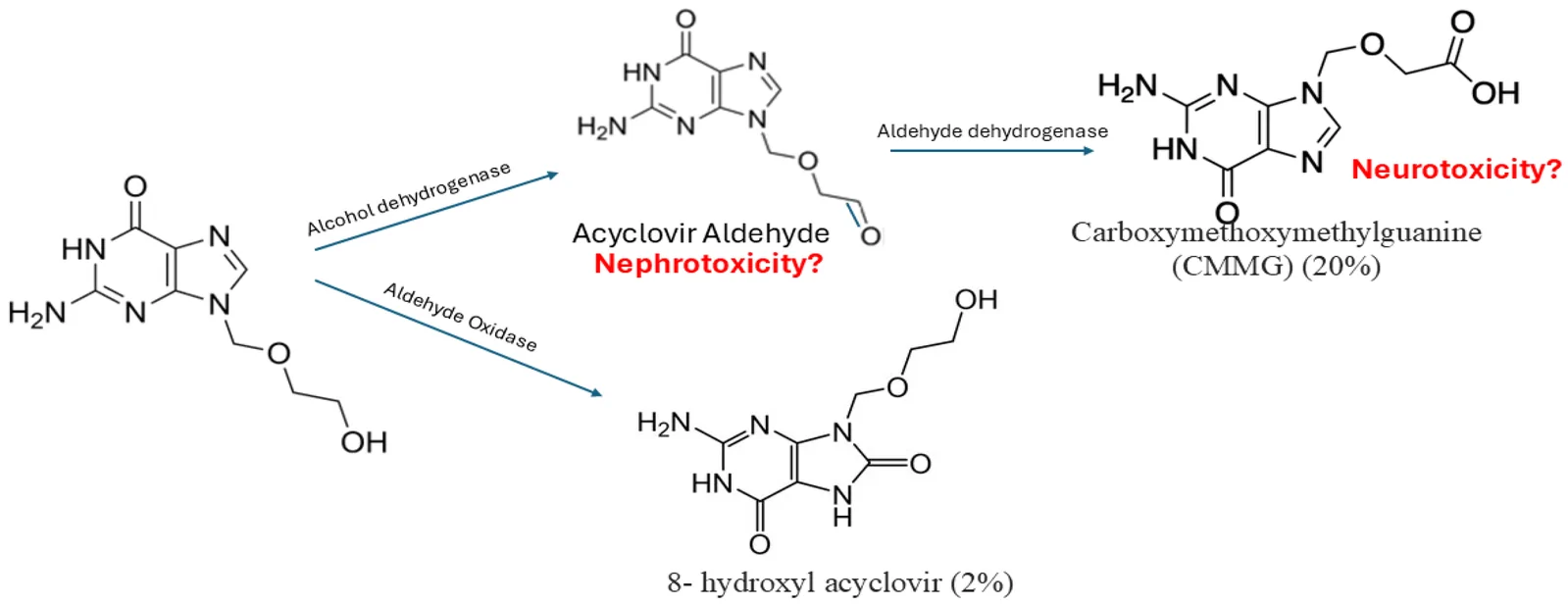
Acyclovir metabolism. Acyclovir is primarily metabolized by alcohol dehydrogenase to form acyclovir aldehyde, which is subsequently converted by aldehyde hydrogenase into 9-carboxymethoxymethylguanine. Additionally, a smaller portion is metabolized by aldehyde oxidase to produce 8-hydroxy acyclovir. Carboxymethoxymethylguanine is suggested to be associated with neurotoxicity, while acyclovir aldehyde is suggested to be associated with neurotoxicity, yet not confirmed.

Acyclovir is metabolized in the kidney through sequential steps by ADH to acyclovir aldehyde, and then acyclovir aldehyde is further metabolized by ALDH to form CMMG. And oxidized by aldehyde oxidase to form 8-hydroxyl acyclovir [12]. Around twenty percent of acyclovir is metabolized to CMMG, 2% is metabolized to 8-hydroxyl acyclovir, and the remaining acyclovir is excreted unchanged in the urine [41]. CMMG and 8-hydroxy acyclovir are recovered unchanged in the urine [42]; however, it is not well studied in the literature whether a transporter is involved in their elimination. Patients with impaired renal function had higher percentages of metabolites excreted in the urine [41]. CMMG is primarily eliminated through the kidney and has a half-life of around 1–1.6 h in cynomolgus monkeys [12] and a time to maximum concentration of 3.3 ± 0.52 h [41]. The process of acyclovir metabolism is presented in Figure 2.

## 4. Pharmacological and Toxicological Profile of the Metabolites

Although acyclovir metabolites are suggested to be pharmacologically inactive, the toxicity of acyclovir is not known, whether it is caused by it or attributed to its metabolites; see Figure 1B. An opinion is that acyclovir could lose selectivity in high concentrations, causing inhibition of mitochondrial DNA polymerase, which leads to toxicity [43,44]. However, recent studies are showing the potential association between acyclovir-induced toxicity and its metabolites.

Acyclovir-induced nephrotoxicity is serious and requires great attention. It is well reported that acyclovir crystallization in the kidney is the main cause of nephrotoxicity. However, some studies looked at the effect of acyclovir metabolites on the kidney, which could be adding a more toxic effect to the kidney tissues. The acyclovir aldehyde intermediate formed before CMMG could be playing a role in the nephrotoxicity through direct insult to the kidney. Most aldehydes are reactive and toxic and contribute to hepatotoxicity and nephrotoxicity through different mechanisms [45]. Blocking the first step of the conversion of acyclovir to acyclovir aldehyde led to a decrease in the nephrotoxicity [17]. However, this was only reported by a single in vitro study, and it is not well understood whether blocking the metabolism of acyclovir could prevent its toxicity or not. An in vitro model of human renal proximal tubular (HK-2) cells tested the expression of acyclovir-metabolizing enzymes on these cells and the toxicity of acyclovir metabolites. They found that acyclovir-metabolizing enzymes are present in HK-2 cells. Moreover, they found that acyclovir aldehyde produced by ADH has an impact on cell death, but not CMMG, which is produced by ALDH2 [17]. The blockage of the formation of the aldehyde metabolite led to a decrease in cell death. Moreover, a proteomic characterization of acyclovir-induced nephrotoxicity in mice reported that in the kidney tissue of the acyclovir-treated group, there was evidence of oxidative and mitochondrial stress. Increased expression of crystallin alpha B and peroxiredoxin in the acyclovir-treated group suggests activation of protective antioxidant responses. However, reduced expression of fibroblast growth factor indicates suppression of regenerative signaling, which may impair tissue repair. The decrease in cytochrome c oxidase points to mitochondrial dysfunction and possible energy deficits or activation of cell death pathways. Overall, these findings suggest a mixed response, with partial cellular adaptation but potential early signs of acyclovir-induced nephrotoxicity [46]. As acyclovir aldehyde is a hetero-aromatic intermediate aldehyde formed inside the renal tubular cells, looking generally into aldehyde toxicity could be useful in pointing out its association with acyclovir-induced nephrotoxicity. One of the possible mechanisms by which aldehydes cause toxicity is that aldehydes are electrophiles in nature and could form adducts with nucleophiles such as DNA and proteins [47]. Another mechanism is the toxicity through the formation of reactive oxygen species and depletion of antioxidants, which in turn leads to oxidative stress [45]. Moreover, aldehydes could cause toxicity to the mitochondria through several pathways [48]. This concept raises the question: Does blocking the acyclovir metabolism in the kidney prevent nephrotoxicity? Further studies are needed to answer this question.

Few studies and case reports have reported that CMMG “acyclovir metabolite” concentration is positively correlated with the neurotoxicity symptoms [20,49]. The exact mechanism is unknown, given the small number of cases of neurotoxicity and the difficulties in exploring this type of toxicity in humans. Obembe et al. recently reported that high acyclovir dose impairs motor function, muscle strength, and memory by reducing anti-inflammatory cytokine (interleukin-10) and antioxidant (catalase, superoxide dismutase, and glutathione) and increasing malondialdehyde and pro-inflammatory cytokines (IL-6 and TNF-α) in rats. Moreover, acyclovir increased serotonin levels and decreased dopamine levels in the brain tissues of rats treated with a high acyclovir dose. Both dopamine and serotonin are monoamine neurotransmitters that regulate homeostatic processes like mood, sleep, and appetite, with dopamine primarily mediating motivational salience and reward-seeking behavior, while serotonin modulates emotional processing. The imbalance between dopamine and serotonin could lead to mental and emotional disturbance. However, they did not correlate these findings with either acyclovir or CMMG concentrations [50].

While the physical obstruction of renal tubules is well-documented as a cause of nephrotoxicity, a possible target of acyclovir toxicity may involve the direct injury to renal tubular cells. Furthermore, the neurotoxic metabolite CMMG is hypothesized to act as a metabolic disruptor during states of systemic accumulation in the central nervous system. There is limited evidence of acyclovir metabolites’ contribution to acyclovir-induced toxicity and the underlying mechanism of toxicity. Therefore, more studies are required to prove the association between acyclovir metabolites and toxicity to provide possible strategies to avoid the toxicity.

## 5. Risk Factors Associated with Acyclovir-Induced Toxicity

In the literature, some studies evaluated the correlation between several factors and acyclovir-induced toxicity. Age, concomitant nephrotoxic medications, overdose, and renal dysfunction are found to be associated with increased toxicity [19,43,51,52]. The acyclovir dose and the rate of administration are the main factors that contribute to toxicity. Receiving a higher than the recommended dose was the only factor associated with acyclovir-induced nephrotoxicity, as reported by Aboelezz et al. The main reason for administering a high acyclovir dose in that study was the body weight used to calculate the dose, especially in obese patients (as acyclovir dosing is weight-based) [19]. If obese patients receive acyclovir based on their actual body weight, they are at a very high risk of developing toxicity [53,54,55].

Kidney function is also a very crucial factor that should be considered, as patients with impaired kidney function are highly prone to acyclovir-induced toxicity [43,56]. Therefore, patients with reduced kidney function should receive an adjusted dose. Moreover, age itself is considered a risk factor for chronic kidney disease; therefore, attention should be given to it while prescribing acyclovir [51]. Another factor that could greatly affect acyclovir-induced toxicity is the fluid balance of the patient while on acyclovir therapy. Although fluid balance was one of the factors that is not well captured due to the limited and retrospective nature of most of the published studies, it is recommended that patients are well hydrated while on acyclovir. A proposed hypothesis is that dehydration leads to a reduced rate of urine flow, and acyclovir concentrates in the renal tubules with crystal formation [55].

Concomitant medications could also significantly heighten the risk of acyclovir toxicity through two main pharmacological mechanisms. Firstly, co-administration with other nephrotoxic drugs (e.g., vancomycin, aminoglycosides, nonsteroidal anti-inflammatory drugs) increases the overall risk of acyclovir-induced toxicity, which subsequently impairs the kidney’s ability to clear acyclovir, leading to toxic accumulation. Secondly, drugs like probenecid that compete with acyclovir for the same organic anion transporter systems in the renal tubules block the secretion of acyclovir. This competitive inhibition directly decreases acyclovir’s renal clearance, resulting in elevated and potentially toxic plasma concentrations. Therefore, clinicians must carefully review a patient’s entire medication list and try to minimize the chance of toxicity when combining acyclovir with these specific drug classes.

## 6. Clinical Manifestation of Metabolite Toxicity

It is well known that high levels of acyclovir cause toxicity; however, the causation of the metabolites with toxicity is not well studied in humans. Some studies measured the levels of the metabolites and tested their correlation with acyclovir-induced toxicity.

### 6.1. Neurotoxicity

Although the prevalence of neurotoxicity in the literature is not reported, it has a mean onset of 3.1 days ± 4.3 days [43]. Neurotoxicity is usually manifested as disorientation, decreased level of consciousness, hallucination, agitation, and others [43]. Diagnosis of neurotoxicity is challenging in some cases, as those who are receiving acyclovir to treat herpes encephalitis. However, the clinical scenario could guide us to diagnose acyclovir-induced neurotoxicity, and if available, acyclovir and CMMG levels could be confirmatory [57,58]. To differentiate these conditions clinically, clinicians should be vigilant in identifying patients with impaired renal function who develop sudden visual hallucinations, slurred speech, or myoclonus shortly after starting treatment, which strongly suggests acyclovir-induced neurotoxicity. In contrast, herpes encephalitis typically presents with a high fever, seizures, and focal personality changes that emerge regardless of kidney function and usually precede the start of medication. While toxicity improves rapidly once the drug is stopped or cleared via dialysis, encephalitis will worsen without continued high-dose treatment and is usually confirmed by finding abnormalities on a brain MRI or elevated white blood cells in the spinal fluid. A systematic review of cases conducted in 2021 found 119 cases of acyclovir and valacyclovir-induced neurotoxicity in the literature. Although it could be a relatively small number given the frequent usage of acyclovir, it highlights the importance of clearer guidance to avoid acyclovir neurotoxicity [43]. The concentrations of acyclovir and its metabolite (CMMG) in the serum above 100 µmol/L and 10.8 µmol/L, respectively, were suggested to contribute to toxicity [20,59]. While in cerebrospinal fluid (CSF), CMMG levels above 1 µmol/L are linked to neurotoxicity. The toxicity was resolved with one or more of these approaches: stopping acyclovir, adequate hydration, or reducing the dose [60,61]. Clinicians should be vigilant that not only acyclovir may cause neurotoxicity, but also valacyclovir still has the potential to cause toxicity. Table 1 summarizes reports of acyclovir-induced neurotoxicity and associated CMMG and acyclovir levels. All the patients with neurotoxicity had a parallel nephrotoxicity except one patient [49]. In neurotoxicity, elevated CMMG levels could be accompanied by elevated acyclovir levels [20]; however, in some cases, only CMMG is elevated. Current evidence identifies elevated CMMG levels as a key mediator in the development of acyclovir-induced neurotoxicity [49,62].

Around 25% of acyclovir is converted to CMMG in renal impairment patients, compared to 12.6% in patients with no renal impairment [18]. Usually, nephrotoxicity precedes neurotoxicity, which is why more accumulation of acyclovir and CMMG could end with neurotoxicity. However, there are reports that neurotoxicity could occur without nephrotoxicity. For instance, a case report of a 63-year-old male who experienced acyclovir-induced neurotoxicity despite normal kidney function (serum creatinine 1.2 mg/dL and glomerular filtration rate 60 mL/min). The patient returned to normal neurological function after stopping acyclovir [63]. Few studies recommend therapeutic drug monitoring of acyclovir and CMMG to ensure therapeutic concentrations [18,61,64]. While a definitive “causal” link in human models is difficult to prove, the overwhelming correlation between CMMG levels and neuropsychiatric symptoms suggests that it could be the primary neurotoxic mediator. However, this does not negate that there could be other possible causes that are accompanied by elevated CMMG levels. This highlights the knowledge gap of the mechanisms by which acyclovir induces neurotoxicity, and additional research is required to clarify the mechanism by which CMMG may induce neurotoxicity, if it plays a role.

**Table 1:** Acyclovir-induced neurotoxicity and associated CMMG and acyclovir levels.

Author, Year [Reference]	Study Design	Age (Years)	Sex n (%Male)	Acyclovir Dose	Day of Neurotoxicity	Serum Acyclovir µmol/L	Serum CMMG µmol/L	CSF Acyclovir µmol/L	CSF CMMG µmol/L	Baseline CrCl mL/min/1.73 m^2^	Associated Nephrotoxicity? mL/min/1.73 m^2^
Berry et al., 2014 [60]	Case report	75	Female	10 mg/kg every 8		182.2	57.3	0.88	1.6	78	Yes, 20
Lee et al., 2012 [61]	Case report	63	Female	Valacyclovir 2 gm q8h	3	NR	NR	NR	3.35	85 mL/min, creatinine 65 μmol/L	Yes, creatinine 513 μmol/L
Vonberg et al., 2023 [65]	Case report	70	Female	NR	2	NR	88.7	NR	NR	NR	NR
Yang et al., 2007 [62]	Case report	70	Male	500 mg daily		7.1	20.9	NR	NR	6.2 mg/dL	Chronic end stage kidney disease (hemodialysis) 5.7 mg/dL
Helldén et al., 2006 [49]	retrospective study, 21 (neurotoxicity *n* = 9)	54 ± 14 ^b,e^	7 (77.8) ^b,e^	Oral 1600–6000 mg/day IV 250–3750 mg/day	NR	NT 22.6 (1.4–97.9) no NT 8.1 (3.5–14.6)	NT: 27.6 (6–161) No NT: 0.8 (0.5–1)	NR	Neurotoxicity1 (0.6–7) No neurotoxicity < 0.5	72.0 ± 25.4 mL/min	8 patients out of 9 Neurotoxicity *n* = 8: 15 ± 9 mL/min No neurotoxicity *n* = 1: 87 ± 22 mL/min
Helldén et al., 2003 [20]	observational study, 93 (neurotoxicity *n* = 49)	59 ± 15 ^c,e^	25 (51) ^c,e^	Oral acyclovir 1780 ± 1290 Oral valacyclovir 1450 ± 725 IV acyclovir 1294 ± 1095	NR	NT: 21.0 ± 30.7 no NT: 7.2 ± 6.7	NT 34.1 ± 39.4 no NT 4.7 ± 4.7	NR	NR	19.5 ± 21.1 mL/min	They are chronic kidney disease patients
Lindström et al., 2019 [66]	Prospective observational, 21 (neurotoxicity *n* = 5)	59 ± 13.9 ^d,e^	2 (40) ^d,e^	14 (10–15)		NT ^a^ 50 (15–110) no NT 13 (5.7–75)	NT 9.7(3.8–57) no NT 1.6 (0.7–4.9)	27 (16–40)	3.3 (0.29–6.4)	NR	Yes

Data are represented as mean ± standard deviation or median (IQR) unless otherwise specified. In the case of the observational study, the presented data are only for patients who had acyclovir-induced neurotoxicity. ^a^ based on day 1 sampling; ^b^ *n* = 9; ^c^ *n* = 49; ^d^ *n* = 5; ^e^ patients with neuropsychiatric symptoms. Abbreviations: CMMG, carboxymethoxymethylguanine; CSF, cerebrospinal fluid; NR, not reported; NT, neurotoxicity.

### 6.2. Nephrotoxicity

The prevalence of acyclovir-induced nephrotoxicity has been reported in the literature to range from 6% to 21% [19,53,56], with a mean onset of 2 days (range: 1–14) after the initiation of acyclovir therapy [56]. Nephrotoxicity is manifested as acute kidney injury, where the patient could experience one or more of the following: decreased urine output, elevated blood urea nitrogen and serum creatinine levels, nausea, and vomiting [67,68]. It is well reported in the literature that high acyclovir concentrations crystallize in the kidney and cause obstructive nephropathy, which necessitates adequate hydration while on acyclovir therapy [69]. Moreover, limited evidence suggests that acyclovir aldehyde, an intermediate that is converted to CMMG, could have a role in direct renal tubular toxicity [17]. Acyclovir-induced nephrotoxicity is usually reversible with proper management. If acyclovir-induced nephrotoxicity is left untreated, the patient may deteriorate rapidly, and acyclovir-induced neurotoxicity could happen.

## 7. Induction or Inhibition of ADH and ALDH

There is a lack of evidence on the effect of ADH and ALDH inducers and inhibitors on acyclovir metabolism. Although acyclovir metabolism plays a minor role in its elimination, the effect of these enzyme inducers and inhibitors should be studied, as acyclovir metabolites are potentially toxic. Since ADH metabolizes acyclovir to acyclovir aldehyde and ALDH further oxidizes it to CMMG, evaluating the induction of these enzymes may provide insights into the potential nephrotoxicity and neurotoxicity associated with acyclovir aldehyde and CMMG, respectively [12,17]. To illustrate, chronic and acute alcohol consumption could differently disturb the ratio of ADH/ALDH, which in turn affects the levels of metabolites formed [70,71]. Examples of drugs that inhibit ADH are fomepizole and cimetidine, while disulfiram and cyanamide inhibit ALDH [72,73]. However, the inhibitory effect was studied on the liver, and only a few studies are available on the kidney enzymes. For instance, ethanol consumption for 30 weeks resulted in around a 71% increase in alcohol dehydrogenase in rat kidneys [74]. Moreover, the presence of the ALDH genetic polymorphism, the ALDH2*2 allele, has been reported to cause acetaldehyde intolerance due to inhibition of ALDH activity [72]. This may be extrapolated to the metabolism of acyclovir; however, further studies are needed to confirm this. Furthermore, in 18 Japanese hemodialysis individuals, ALDH2 polymorphism affected acyclovir elimination half-life but not that of valacyclovir and CMMG. The presence of ALDH2*2/*2 resulted in the longer elimination half-life of acyclovir [75]. Further studies on the effect of ADH and ALDH inducers and inhibitors are needed to examine the effect of their inducers and inhibitors on acyclovir metabolism.

## 8. Management and Prevention Strategies

Acyclovir-induced toxicity is a serious health concern and requires immediate medical attention. It is essential for clinicians to maintain a high degree of awareness of acyclovir-induced toxicity and prevent it or early detect and manage it accordingly. Although there are no direct cost analysis studies of the economic burden of acyclovir-induced toxicity, it increases health care costs. The increased costs could be attributed to longer hospital stays, costs of hemodialysis, the extensive use of health resources, and the sequences of acyclovir discontinuation in patients with serious conditions [43,62]. Some of the preventative measures that are suggested to prevent acyclovir toxicity are slow acyclovir infusion for at least an hour, adequately hydrating the patient, and administering the appropriate dose [19,76]. When the patient experiences toxicity, acyclovir might be discontinued or the dose reduced, or the patient might need hemodialysis. Acyclovir is the first-line therapy to treat herpes infections; therefore, ensuring an effective dose without exposing the patient to toxicity is important. In the case of herpes encephalitis, alternatives to acyclovir are not well studied. However, a case report showed that ganciclovir could be an antiviral in the case of herpes encephalitis [77]. Famciclovir could be an alternative to acyclovir to treat genital herpes, with dose adjustment according to kidney function [78].

### 8.1. Dose Adjustment

As acyclovir is mainly eliminated through the kidney, any nephrotoxicity necessitates dose reduction, prolonging the dosing frequency, or even stopping acyclovir in very severe cases. Adequate hydration at this point is crucial to prevent or facilitate the elimination of acyclovir crystals formed in the kidney tubules. Moreover, acyclovir half-life is greatly increased in renal dysfunction, so normal acyclovir dosing could lead to rapid acyclovir accumulation.

### 8.2. Dialysis

Acyclovir is a dialyzable drug, so dialysis could be a good option if a rapid reduction in acyclovir concentrations is required. Hemodialysis could be a viable and effective option, as acyclovir has a relatively low molecular weight of 225 g/mol, low plasma protein binding, and high hydrophilicity. Hemodialysis is usually an option in cases of severe toxicity.

### 8.3. Therapeutic Drug Monitoring

Monitoring acyclovir and CMMG levels could be a good tool to prevent or early manage acyclovir-induced toxicity. A prospective observational study suggested that monitoring acyclovir and CMMG levels, CSF to serum albumin ratio, and renal function could be helpful tools to predict acyclovir-induced neurotoxicity. They have found that CMMG was significantly higher in the serum and the CSF of patients who developed neurotoxicity [66]. Few labs in Sweden currently measure acyclovir and CMMG levels as a therapeutic drug monitoring of patients while on acyclovir therapy. This helped them to differentiate acyclovir-induced neurotoxicity from other neurological conditions, with a median (range) of 33 (17–59) patients per year over a study period of 10 years. They recommended that acyclovir and its metabolite blood level measurements should be a part of the standard care to help diagnose neurotoxicity and avoid further health care costs [79]. A recently published method of analyzing acyclovir and CMMG simultaneously applied their method to a sample of a patient experiencing neurotoxicity. The acyclovir concentration was 8.2 mg/L, while the CMMG concentration was 8.5 mg/L [80]. Sometimes it is difficult to differentiate between acyclovir-induced neurotoxicity and herpes encephalitis. Usually, the patient presents with encephalitis symptoms for which he takes acyclovir. After around 48–72 h, new neurological symptoms could start to appear because of acyclovir-induced neurotoxicity. One of the suggested approaches to confirm neurotoxicity and to start managing accordingly is to monitor serum and CSF levels of acyclovir and CMMG [81].

## 9. Conclusions

Acyclovir metabolites may potentially contribute to its toxicity. Although acyclovir crystal accumulation in renal tubules could lead to obstructive nephropathy, acyclovir aldehyde could cause direct tubular injury. CMMG has been linked with neurotoxicity, yet the exact mechanism is unknown. Based on this review, acyclovir-induced nephrotoxicity is associated with elevated acyclovir levels, which subsequently increase CMMG concentrations and could potentially lead to neurotoxicity.

## 10. Future Directions

While current literature correlates acyclovir metabolites with adverse clinical outcomes, further research is required to transition from correlation to causality. Future studies should employ in vitro and in vivo models to isolate and examine the cytotoxic profiles of acyclovir aldehyde and CMMG independently. This would help determine if these molecules act as direct toxins or merely as biomarkers of impaired renal clearance. Furthermore, the enzymatic pathways involving alcohol dehydrogenase (ADH) and aldehyde oxidase (ALDH) present a potential therapeutic target. Investigating whether the isoform-specific inhibition of ADH prevents the formation of acyclovir aldehyde could clarify its specific role in nephrotoxicity. Similarly, exploring whether blocking the conversion of the aldehyde intermediate into CMMG, the primary candidate for neurotoxicity, limits neuropsychiatric symptoms in renal impaired models is a required area for investigation. If the causation is proven, such metabolic modulation could eventually lead to strategies designed to maximize antiviral efficacy while minimizing metabolite systemic toxicity.

## Data Availability

The original contributions presented in this study are included in the article. Further inquiries can be directed to the corresponding author.

## Abbreviations

The following abbreviations are used in this manuscript:

ADH	alcohol dehydrogenase
ALDH2	aldehyde dehydrogenase 2
BCRP	human breast cancer resistance protein
CMMG	carboxymethoxymethylguanine
CSF	cerebrospinal fluid
HK-2	human renal proximal tubular
HSV	Herpes simplex virus
IV	intravenous
OATs	organic anionic transporters
VZV	Varicella zoster virus
